# Evolution of the intermuscular bones in the Cyprinidae (Pisces) from a phylogenetic perspective

**DOI:** 10.1002/ece3.5374

**Published:** 2019-07-17

**Authors:** Kunfeng Yang, Wansheng Jiang, Xiaoai Wang, Yuanwei Zhang, Xiaofu Pan, Junxing Yang

**Affiliations:** ^1^ Yunnan Key Laboratory of Plateau Fish Breeding, Kunming Institute of Zoology Chinese Academy of Sciences Kunming China; ^2^ State Key Laboratory of Genetic Resources and Evolution, Kunming Institute of Zoology Chinese Academy of Sciences Kunming China

**Keywords:** character evolution, Cyprinidae, ecological adaptation, intermuscular bones, X‐rays

## Abstract

Intermuscular bones (IBs) are widely present in morphologically generalized teleost fishes and are commonly found in the Cyprinidae. Intermuscular bones are small, hard spicules of bone that are formed by ossification in the myosepta between neighboring myomeres. Why fish have IBs, and whether there is any evolutionary pattern to their occurrence, has been poorly understood. However, the presence of IBs does substantially affect the meat quality and commercial values of many cyprinid fishes in aquaculture. In this study, we sampled 592 individuals of cyprinid fishes to systematically investigate the evolution of IBs from a phylogenetic point of view. We found that the total number of IBs in the Cyprinidae ranged from 73 to 169, and we clarified that only two categories of IBs (epineural and epipleural) were present in all examined cyprinids. Most of the IBs were distributed in the posterior region of the fish, which might be an optimal target for selecting fewer IB strains in aquaculture. There was a positive correlation between IBs and the number of vertebrae, thus making it possible to predict the approximate number of IBs by counting the number of vertebrae. Although the IBs displayed some correlation with phylogenetic relationships in some lineages and to ecological factors such as diet (especially carnivore), in an overall view the variations of IBs in cyprinids were extremely diverse. The number and patterns of IBs in these fishes may reflect their phylogenetic history, but have been shaped by multiple environment factors. In this study, we also confirmed that X‐ray photography remains an optimal and reliable method for the study of IBs.

## 1 INTRODUCTION

Cypriniformes constitute the largest monophyletic group of freshwater fishes on the earth, with 4,000+ species recognized and some 2,000+ species still awaiting description (Mayden et al., 2009; Stout, Tan, Lemmon, Lemmon, & Armbruster, 2016). Containing the vast majority of taxa found in the Cypriniformes, the Cyprinidae is the most speciose family of freshwater teleosts, including more than 3,000 species (Nelson, Grande, & Wilson, 2016). Cyprinids are widely distributed in the freshwaters of the world, including the mainland Eurasia, Japan, most of the East Indian islands, Africa, and North America (Winfield & Nelson, 1991). The habitats of cyprinids are very diverse, covering most freshwater areas, including extreme regions such as some of the world's highest place (e.g., the Qinghai–Tibet plateau), karst caves, and the saline conditions in coastal waters. The members of this family not only exhibit diverse features of evolutionary interest, but also have considerable applied importance to mankind (Winfield & Nelson, 1991). Representative examples include the zebrafish (*Danio rerio*), a model organism used in genomic and developmental biology, important aquaculture species like the common carp (*Cyprinus carpio*), and many popular aquarium species like goldfish (*Carassius auratus*; Stout et al., 2016).

One feature of the Cyprinidae, the intermuscular bones (IBs), has received very little attention from researchers. Intermuscular bones are small bony spicules that are formed by ossifications within the myosepta among neighboring myomeres (Meng, Su, & Li, 1987). They are thus a part of the axial skeleton of the endoskeleton system. Intermuscular bones, which ossify segmentally and are serially homologous in the myosepta, occur only in teleosts among recent vertebrates (Patterson & Johnson, 1995). Almost all species of cyprinids have IBs. According to the position of attachment in fish muscle, IBs have been subdivided into three categories, namely epineural, epicentral, and epipleural (Owen, 1866). Epineurals are those IBs attached proximally to the neural arches above the horizontal septum, whereas epipleurals are attached to the hemal arches or the ribs that lie below the horizontal septum. Epicentrals attach to the central vertebrae in the plane of the horizontal septum (Owen, 1866). The IBs are of variable occurrence; for instance, in some teleosts, only epineurals and epipleurals are present, and in some Perciformes, IBs have completely disappeared (Patterson & Johnson, 1995). According to the nature of the branch patterns, IBs have been divided into seven types as follows: nonforked, one‐end‐unequal‐bi‐forked, one‐end‐equal‐bi‐forked, one‐end‐multi‐forked, two‐end‐bi‐forked, two‐end‐multi‐forked, and tree‐branch forking (Lv, Bao, Jiang, Yang, & Li, 2007).

Techniques for studying IBs are mainly from anatomy (dissection), clearing and staining, and radiography. All these methods have both advantages and disadvantages and are applicable for different purposes. Dissection has been the most commonly used method for studying IBs in the past few years, as it is an easy and straightforward method of getting results (Andria‐Mananjara, Rasamoelina, & Vandeputte, 2016; Dong et al., 2006; Patterson & Johnson, 1995). However, the tiny IBs in some small individuals can be easily missed. Staining the fish bones using Alizarin red is an alternative method to observe IBs. It is suitable for most of the individuals including small ones as it can retain as much information as possible (Yao, Lv, Gong, Wu, & Bao, 2015); however, the complicated procedure of staining fish bones requires considerable times and is awkward for large individuals. In contrast to the above two more traditional methods, radiographing (usually X‐ray) of specimens to observe IBs is a relatively new method which has a lot of advantages, for example, it is easy to operate and time‐saving, and there is no damage to the specimen; it is especially applicable to living fishes (Perazza et al., 2017; Vallod & Arthaud, 2009; Xu, Zheng, Qian, & Luo, 2015). One of the supposed drawbacks for radiographing is the presence of overlapping areas which could result in misreading the number of IBs when taking radiograph from a single angle; however, this effect has not been evaluated as yet.

There are not very many studies involving IBs, and most of these involve only a few aspects. Many of the papers simply count and describe the IBs based on a few different species (e.g., *Hypophthalmichthys molitrix*, *H. nobilis*, and *Megalobrama amblycephala* in Dong et al., 2006). Although seven types of IBs based on shapes have been identified, all six of the more complex shapes were thought to be evolved from the simple nonforked shape. Intermuscular bones found in the more anterior parts of the body were usually more complex than those present in the posterior section of the fish (Li et al., 2013). Some studies reported that the number of IBs did not increase with growth as a function standard (SL) or total (TL) length and that the number of IBs between the left and right sides of the body fish was not equal in certain individuals (Cao et al., 2014; Li et al., 2013). A recent study pointed out that IBs were probably related to the ploidy of a species; a fish with an increased ploidy would have an increased number of IBs (Li et al., 2013). An interesting pattern concerning the ossification process of IBs has also been reported: The ossification of IBs of most fishes progresses from the posterior to the anterior regions of the body (e.g., *Danio rerio*, Yao et al., 2015, *H. molitri*, Ke, Zhang, Jiang, & Bao, 2009 and *M. amblycephala*, Wan et al., 2014); however, in *Anguilla japonica* the opposite situation is found (Yao et al., 2015). More recent studies focused on the genetic basis of IBs through molecular methods. For instance, Wan et al. (2015) compared the miRNA profiles in *M. amblycephala* and found 20 miRNAs upregulated in IBs relative to connective tissues. A further analysis integrated with the miRNA and mRNA expression profiles has shown that many miRNA‐mRNA interaction pairs might regulate the bone development and differentiation involved in IB formation (Wan et al., 2016).

The real function of IBs is not understood, although several hypotheses have been proposed (e.g., bilateral support for the muscles; Deng, 1959, transmitting muscle strength between sarcomeres; Dong et al., 2006; Lv et al., 2007, or enhancing the strength of herbivorous fishes to some extent; Danos & Staab, 2010). Although the functions of IBs are still largely unknown, the presence of IBs does substantially affect the flesh quality and the commercial values of many aquaculture fishes. Recent studies have shown that IB‐less strains could be obtained by artificial breeding (Perazza et al., 2017; Xu et al., 2015), which shed light on the genetic improvement programs of selecting IB‐less or even IB‐null fishes.

As there are so many questions about IBs awaiting investigations, this study expands our understanding of IBs using representative cyprinid fishes. The main purposes of this study were as follows: (a) to evaluate the reliability of radiographs in counting the number and shape of IBs; (b) to study the number of IBs from a phylogenetic perspective; and (c) to find whether there are any relationships between IBs and potential ecological factors.

## 2 MATERIALS AND METHODS

### 2.1 Sample identifications and selections

A total of 592 specimens of cyprinid fishes were examined in the present study (Table 1). Among them, 88 individuals were fresh samples collected from Yunnan province of China, and the others were ethanol‐preserved samples deposited in the Kunming Institute of Zoology (KIZ), Chinese Academy of Sciences. We identified samples according to the 12‐subfamily classification system of Chen (1998), which is one of the most useful and popular systems for taxonomic studies involving Chinese cyprinids (Jiang et al., 2018). We selected samples for a large coverage of all the 12 subfamilies, and we ensured that each subfamily included at least one genus and two species.

**Table 1:** Sample size of each subfamily of Cyprinidae used in this study

Subfamily name	Genera	Species	Sample size
Danioninae	5	6	43
Leuciscinae	5	5	17
Cultrinae	9	11	70
Xenocyprinae	2	3	19
Hypophthalmichthyinae	1	2	8
Gobioninae	8	13	61
Gobiobotinae	1	5	25
Acheilognathinae	2	4	18
Barbinae	13	43	235
Labeoninae	5	10	38
Schizothoracinae	2	10	37
Cyprininae	4	7	21
Nemacheilidae (outgroup)	1	1	4
Total	58	120	596

### 2.2 X‐ray photographing and counting of IBs

**Figure 1:**
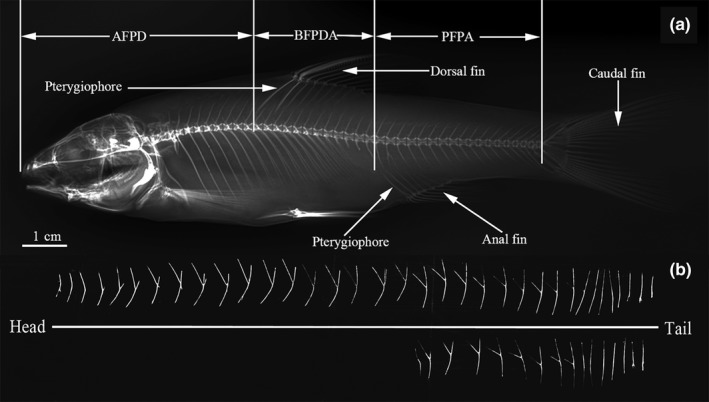
The intermuscular bone (IB) counts from X‐ray photography and anatomy of *Sinocyclocheilus grahami*. (a) lateral view of radiograph with labels dividing regions of a fish; (b) lateral view of IBs that were extracted from a dissected fish

We used the Digital Cabinet X‐ray System (XPERT 80, Kubtec) to scan all the fishes and then saved the X‐ray diagrams. Using the insertional position of the fin pterygiophores in the body of each specimen, the fish was divided into three regions: anterior to the first pterygiophore of the dorsal fin (AFPD), between the first pterygiophore of the dorsal fin and the first pterygiophore of anal fin (BFPDA), and posterior to the first pterygiophore of the anal fin (PFPA; modified from Yang, Pan, Wang, Liu, & Yang, 2015; Figure 1a). For each diagram, we counted the total number of IBs, the epineurals, the epipleurals, and the values for AFPD, BFPDA, and PFPA, using Adobe Photoshop CS4 (Adobe Systems Inc). In order to evaluate the reliability of radiographing in counting IBs, we used 20 individuals of *Sinocyclocheilus grahami* based on the method described above, and then, we dissected these same fishes and counted the IBs for comparisons (Figure 1b).

### 2.3 Determination of fitting curve

In order to find which function model has the best fit to the IB data collected, a fitting curve estimation was employed in SPSS 19 (SPSS for Windows). We selected all function models on the “Models” option to include as much information as possible and then used the parameter of Rsq, a representative of the statistic value of R^2^, to distinguish the best‐fitting curve. The Rsq closest to 1 was identified as the optimal regression model from these various fitting curve categories (such as linear, quadratic, cubic, or inverse). Using the optimal regression model (fitting curve) that was identified from SPSS as the equation category, we drew scatter plots in SigmaPlot 12.5 (SPSS Inc.) and then obtained the specific equation by choosing the optimal curve with *R* ^2^ closest to 1.

### 2.4 Reconstruction of phylogenetic relationship

In consideration of the many polyploid species in Cyprinidae (Yang, Sado, et al., 2015), we reconstructed the phylogenetic relationship using only mitochondrial genes. For each species that we used for IB counting, sequences of three mitochondrial genes (CYT *b*, COI, and ND4) belonging to the species were downloaded from NCBI (Appendix S1). We aligned and trimmed sequences in MEGA6 (Tamura, Stecher, Peterson, Filipski, & Kumar, 2013) and then combined the three gene fragments into a concatenated dataset. The phylogenetic tree was inferred using the Bayesian inference (BI) method with partitioned models in MrBayes 3.1 (Huelsenbeck & Ronquist, 2001). The best model of each gene fragment was calculated using jModelTest version 2.1.6 (Darriba, Taboada, Doallo, & Posada, 2012), under the Akaike information criteria (AIC) as recommended by Posada and Buckley (2004). Posterior probabilities (PP) were based on two independent MCMC runs for 4 × 10^6^ generations, with sampling at every 100 generations yielding 40,000 trees. The first 25% of trees were discarded as burn‐in.

### 2.5 Ancestral reconstruction of the number of IBs

Each species in the phylogenetic tree of the cyprinids was assigned with the total number of IBs that we obtained from radiography. The maximum parsimony method was selected to reconstruct the ancestral state of the total number of IBs using Mesquite 3.10 (Maddison & Maddison, 2016). Then, five separately parallel analyses were performed similar to the ancestral state reconstruction of the total number of IBs, by using the number of two categories of IBs (epineurals and epipleurals) and three regions of IBs (AFPD, BFPDA, and PFPA) as the inputting states.

### 2.6 Correlations of IBs and ecological factors

In order to find whether any correlations between IBs and ecological factors, we further mapped diets, depth of occurrence, and water velocity of these fishes onto the phylogenetic tree. We collected the ecological information from some unpublished sampling records in KIZ, as well as from the literature (Chen, 1998; Chu & Chen, 1989; Cui, Li, & Chu, 2011; Jia, Chen, Tao, & He, 2013; Yan et al., 2012; Yue, Shan, & Lin, 2000; Zeng & Liu, 2009; Zhang, 1998; Zhang & Dai, 2011; Zheng, Chen, & Yang, 2009). According to the food composition of these fishes as adults, we identified three categories of diet in our samples: carnivorous, omnivorous, and herbivorous. The carnivorous fishes predominantly fed on invertebrates and vertebrates, while the omnivores fed on plants, invertebrates, and vertebrates, and the herbivorous fishes mostly took aquatic plants and algae. We also recognized three levels of water depth among these fishes. They are either epipelagic, mesopelagic, or benthic fishes. In some papers, species called pelagic were classified as epipelagic fishes, and middle‐lower ones were reclassified as benthic fishes. Considering that the velocity of water is also an important factor of habitats, these cyprinids were further divided into three classes: lentic (predominantly found in lentic habitats such as lakes and pools), lotic (mostly occurring in streams or rivers), and lentic/lotic fishes (roughly with similar frequency of occurrence in both lentic and lotic habitats).

## 3 RESULTS

### 3.1 Comparative numbers of IBs from radiograph and anatomy

**Figure 2:**
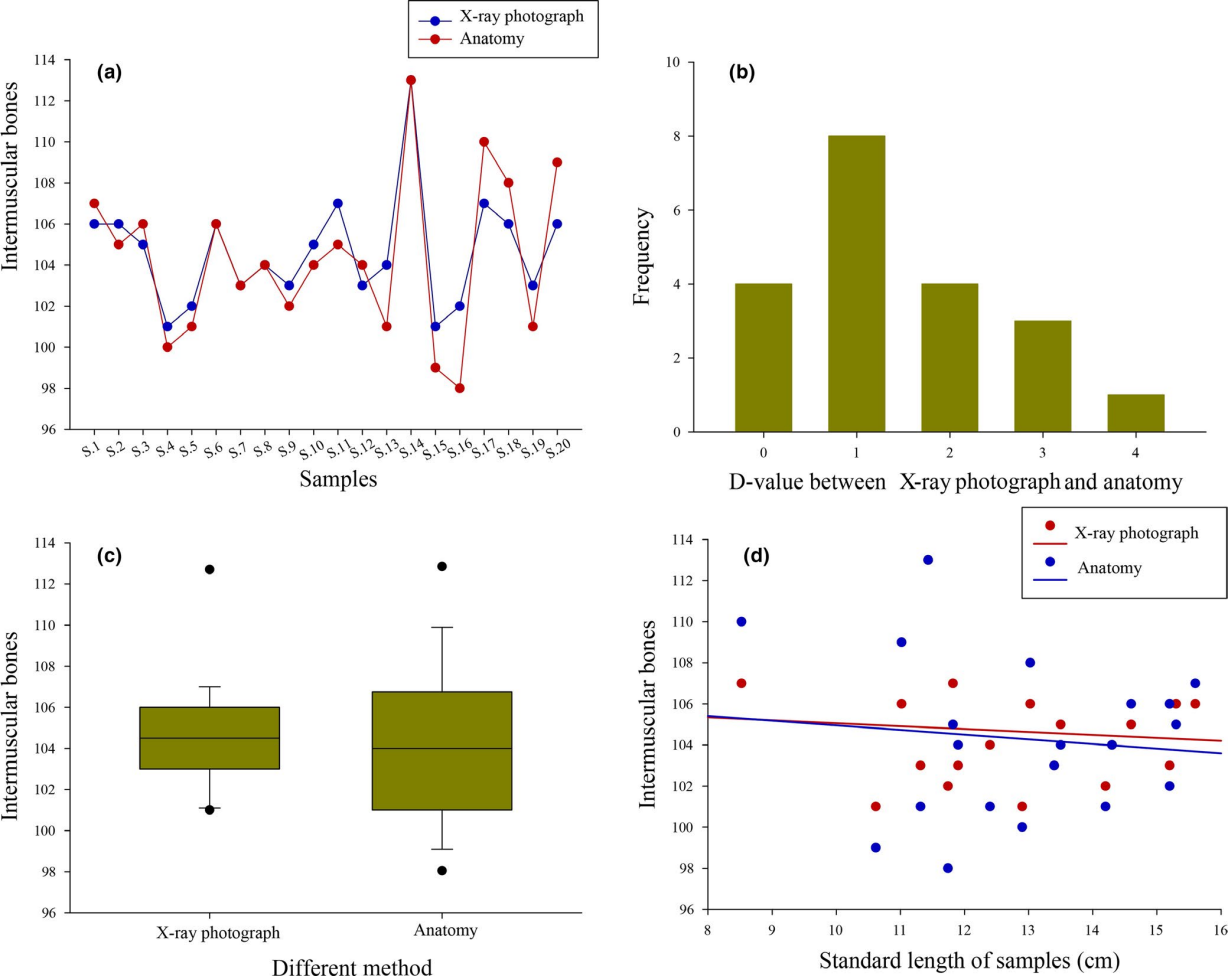
Comparisons of IBs between X‐ray photography and anatomy. (a) quantitative distribution of IBs from each individual; (b) frequency distribution of the deviation value of IBs; (c) the average, maximum, and minimum values of IBs; (d) the number of IBs versus standard lengths

The total number of IBs counted from X‐ray photographs was largely consistent with those obtained using anatomy (Figure 2a). The differences between two counting methods were generally <4, and the variations of 80% of the samples were from 0 to 2 (Figure 2b). The median values of IBs for all samples was 104.6 using X‐ray photograph and 104.3 using anatomy, and the maximum number of IBs was the same (113) in both methods (Figure 2c). In addition, the number of IBs showed no relationship to standard length in either method in the fishes sampled (Figure 2d).

### 3.2 Summary of the number of IBs in Cyprinidae

**Figure 3:**
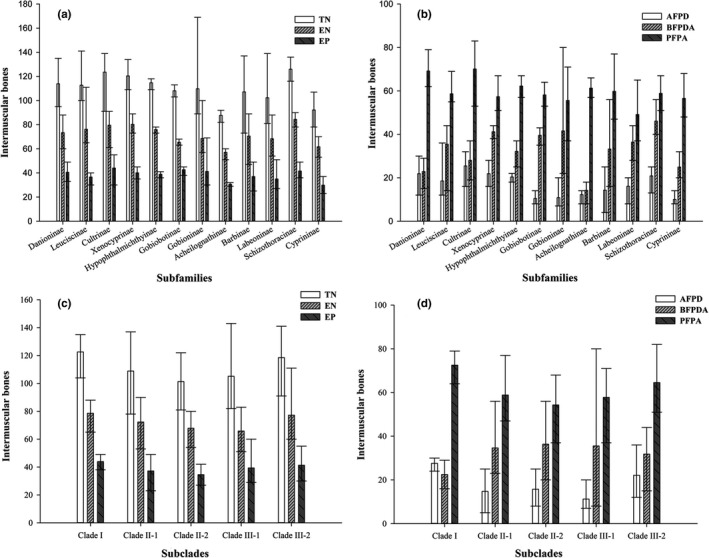
The number of IBs for the two categories and the three regions of the body according to the 12 subfamilies of the Cyprinidae (a, b) and the phylogenetic subclades revealed in this study (c, d). Abbreviations: AFPD: anterior to the first pterygiophore of the dorsal fin; BFPDA: between the first pterygiophore of the dorsal fin and the first pterygiophore of anal fin; EN: epineural; EP: epipleural; PFPA: posterior to the first pterygiophore of the anal fin; TN: total number

In the X‐ray photographs of 592 individuals, only two categories of IBs, the epineural and epipleural, were observed in our specimens. The total number of IBs in cyprinids ranges from 73 to 169, and the average number in each subfamily varies from 88 to 126. The maximum and minimum numbers of IBs were found in the Gobioninae and the Barbinae. The number of epineurals among different subfamilies was generally greater than that of epipleurals. The maximum and minimum numbers of epineurals were present in the Leuciscinae and the Barbinae, while the maximum and minimum epipleural numbers were found in the Gobioninae and the Cyprininae, respectively (Figure 3a).

The number of IBs in different regions of fish body showed that those in the PFPA were greater than those in both AFPD and BFPDA. The number of IBs in PFPA of cyprinids was from 37 to 83, with the average number in each subfamily ranging from 49 to 70. The IBs in AFPD and BFPDA fell within the ranges of 7–36 and 8–80, and these average IBs of each subfamily fell in the ranges of 10–26 and 14–46, respectively (Figure 3b).

### 3.3 Correlation between IBs and vertebrae in Cyprinidae

**Figure 4:**
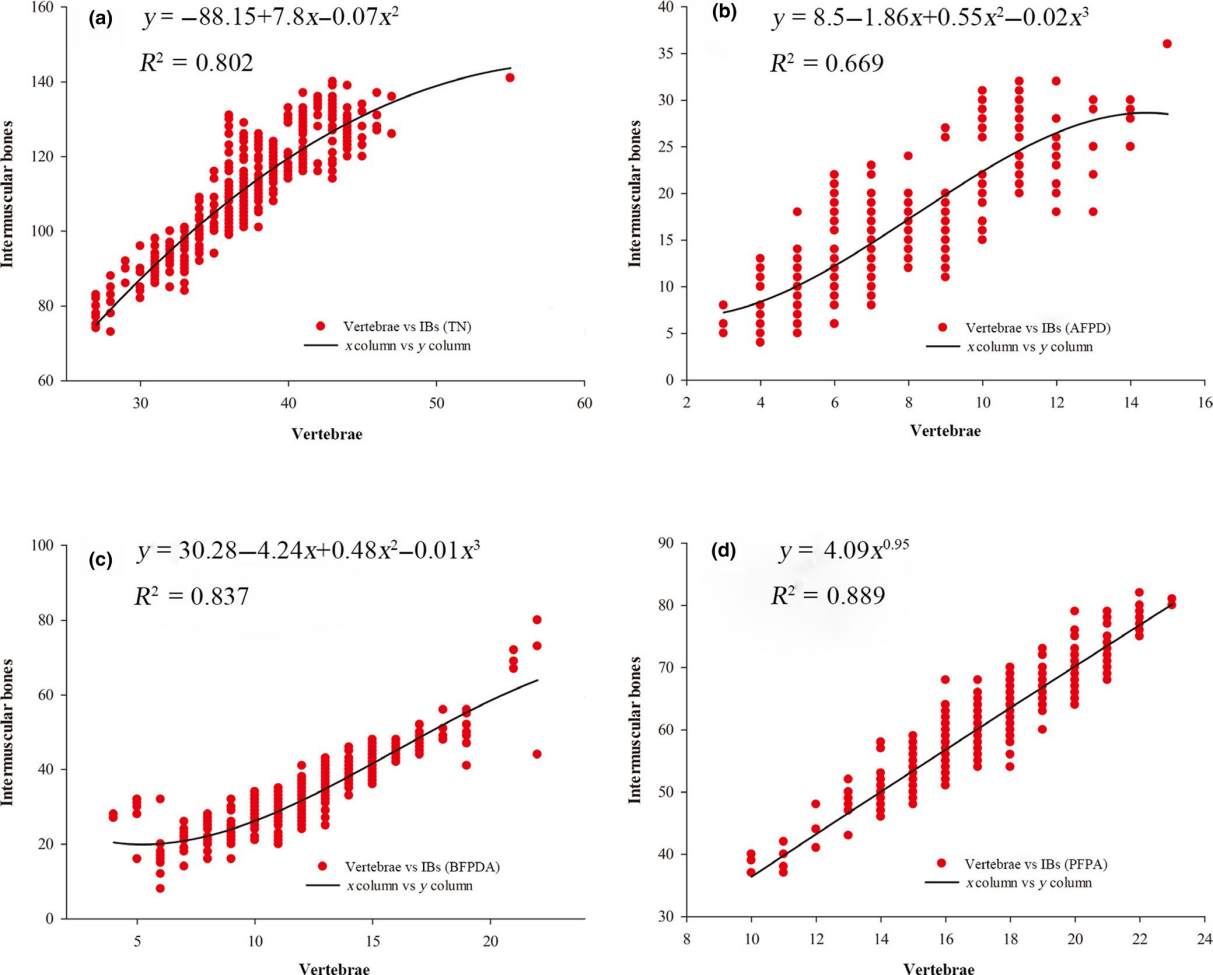
The optimal fitting curve and equation from the scatter plot of IBs and vertebrae. (a) the correlation of total number of IBs and vertebrae; (b) the correlation of IBs at the region of AFPD and vertebrae; (c) the correlation of IBs at the region of BFPDA and vertebrae; (d) the correlation of IBs at the region of PFPA and vertebrae

The number of vertebrae among all studied species in the Cyprinidae falls within the range of 27–55, with an average value of 37. The regression analysis of IBs (*y*) and vertebra (*x*) showed that they fit an optimal equation of *y* = −88.15 + 7.8*x*−0.07*x* ^2^, which indicated a positive correlation (Figure 4). The value of correlation coefficient (*R* ^2^), an indicator of the degree of correlation between the independent and dependent variables (Systat Software, Inc., 2011), was 0.802 (Figure 4).

### 3.4 Phylogenetic relationships of the Cyprinidae in this study

**Figure 5:**
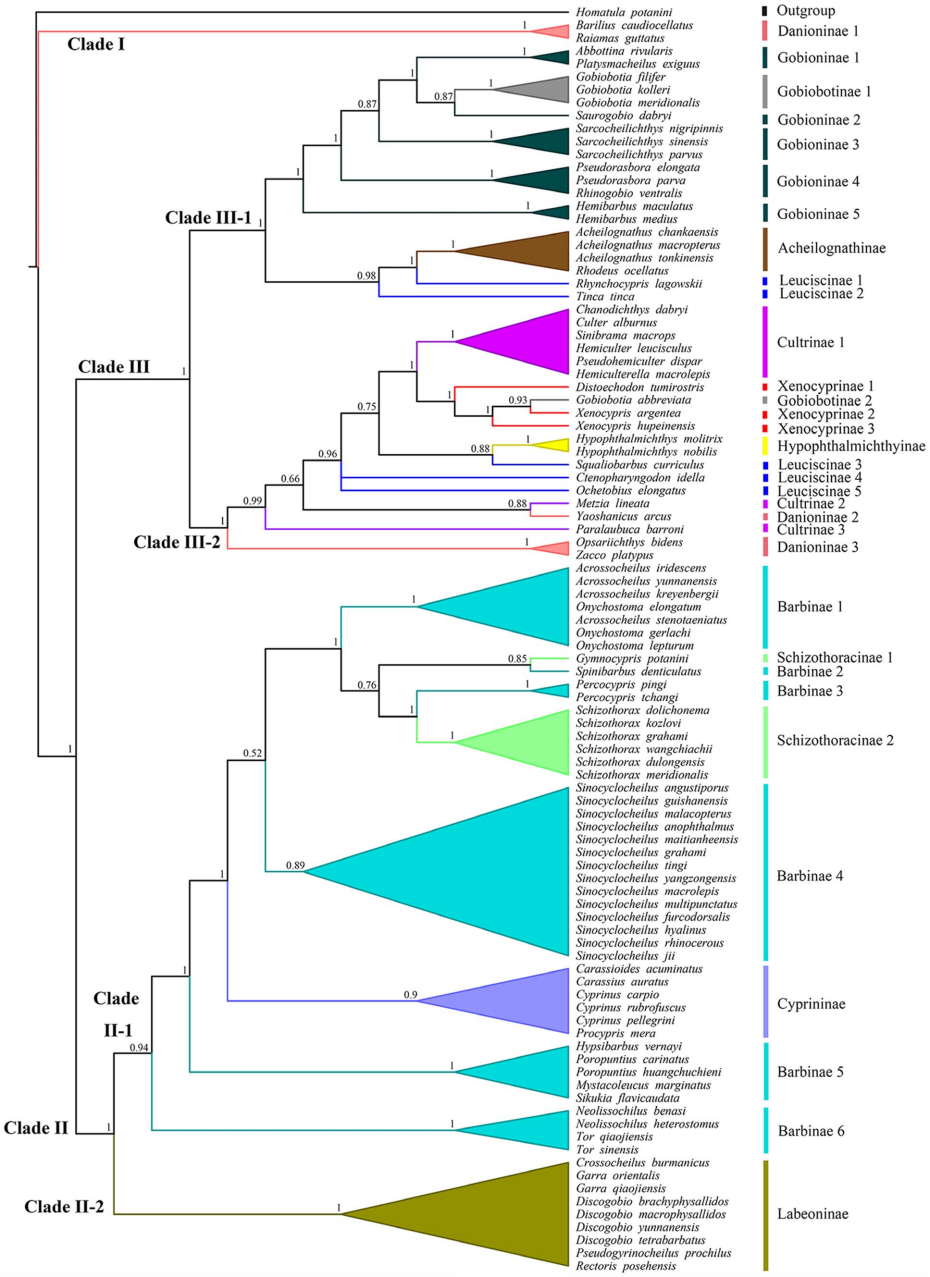
Phylogenetic relationship of Cyprinidae from BI analysis based on a concatenated dataset of three mitochondrial genes (CYT *b*, COI, and ND4). The nodes of species were collapsed to subfamilies (polyphyletic group was assigned with numbers 1, 2, 3…)

A phylogenetic tree of the Cyprinidae based on three mitochondrial genes (CYT *b*, COI, and ND4) revealed three major subclades (Clades I, II, and III) in our analysis (Figure 5). According to Chen (1998), each of the 12 morphologically putative subfamilies was hypothesized to be a monophyletic group based on the skeletal characters elaborated by Chen, Yue, and Lin (1984). Clade I contained two species in the subfamily Danioninae, which was resolved as the basal‐most subfamily, and the remaining cyprinids were divided into two lineages: barbeled cyprinines (Clade II) and nonbarbeled leuciscines (Clade III), with a supporting value of 100%. The lineage of cyprinines (Clade II) contained species in 4 of the 12 recognized subfamilies: Barbinae, Schizothoracinae, Cyprininae, and Labeoninae. However, the Barbinae and Schizothoracinae were not recovered as monophyletic groups. The lineage of leuciscines (Clade III) was further recovered as two subclades (Clades III‐1 and III‐2, PP = 100%); however, these subclades also include many non‐monophyletic morphologically based subfamilies; exceptions are the monophyletic Acheilognathinae and Hypophthalmichthyinae. Many of those non‐monophyletic morphological subfamilies were also revealed by previous molecular studies (Wang, Gan, Li, Mayden, & He, 2012; Yang, Sado, et al., 2015).

### 3.5 Ancestral reconstruction of IBs

**Figure 6:**
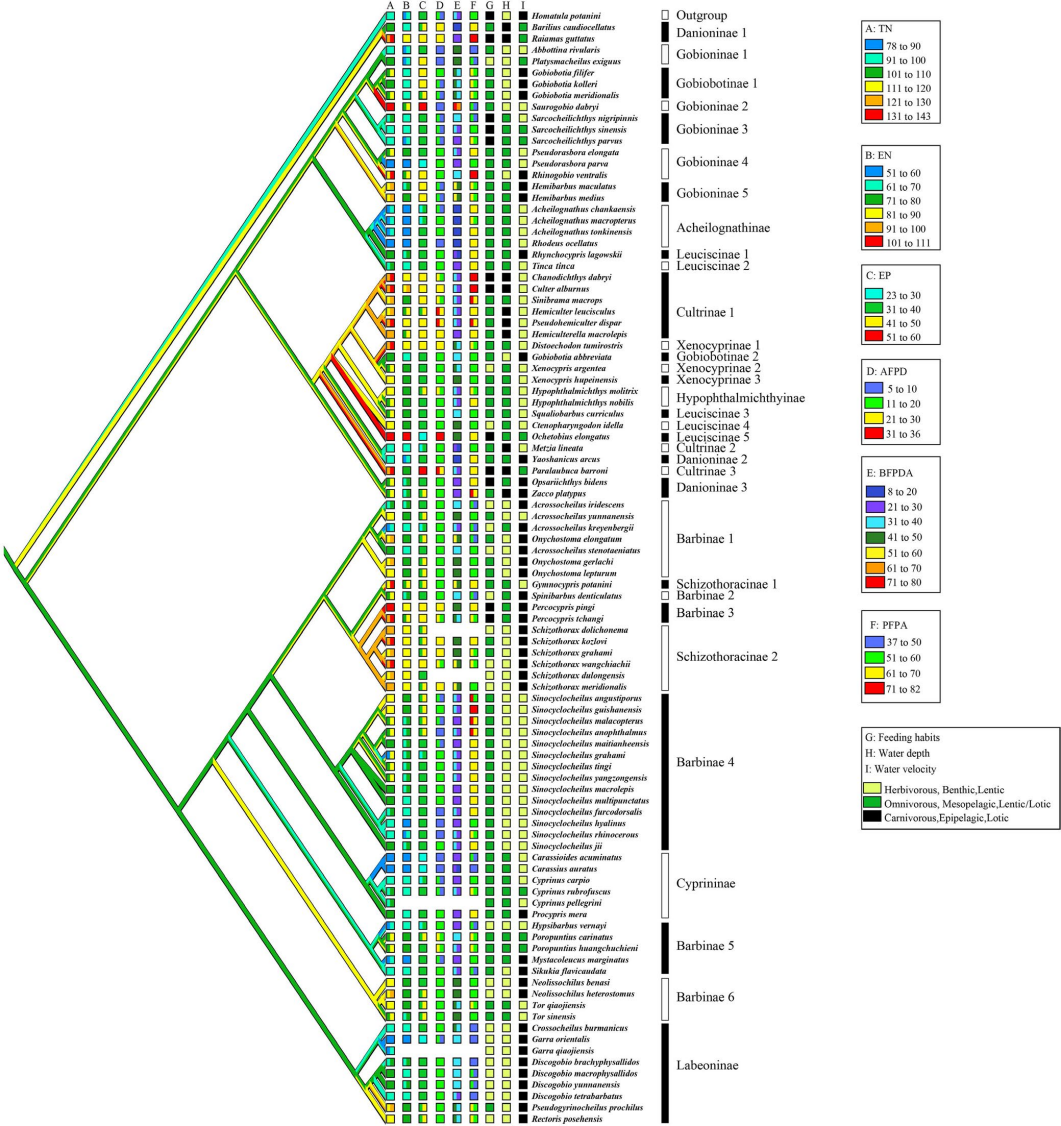
Ancestral state reconstruction of IBs in the Cyprinidae mapped together with three ecological factors. Reconstructed states of (A) total number of IBs, (B) IBs in epineurals, (C) IBs in epipleurals, (D) IBs in AFPD, (E) IBs in BFPDA, and (F) IBs in PFPA. States of three ecological factors including (G) diet, (H) water depth, and (I) water velocity

The ancestral state of the total number of IBs, the two categories of IBs (epineurals and epipleurals), and the three regions of IBs (AFPD, BFPDA, and PFPA) were reconstructed using the phylogenetic relationship of the Cyprinidae fishes that we propose in Figure 5. The ancestral number of IBs of Cyprinidae was calculated to be 101–110, and those numbers varied frequently within the adaptive radiation of cyprinid fishes (Figure 6). The ancestral state reconstruction uncovered some groups, especially those in some monophyletic putative subfamilies, and had similar values to the ancestral number of IBs, such as Hypophthalmichthyinae (109–118), Xenocyprinae (109–134), Leuciscinae (114–141), Cultrinae (119–139), and Gobiobotinae (105–113). For a controversial phylogenetic position of *Tinca* (see more details in Jiang et al., 2018), the number of IBs showed closer relationship to Acheilognathinae than to other Leuciscinae, which was consistent with the current phylogenetic relationship in Figure 5. Another interesting pattern was detected in *Sinocyclocheilus*, the total number of IBs, epineurals, and epipleurals was distinctly varied, and these typical cave fishes (troglobites) had fewer IBs than atypical cave species (troglophiles; terms according to Zhao, Gozlan, & Zhang, 2011). The ancestral number and variation patterns of two categories of IBs (epineurals and epipleurals) and three regions of IBs (AFPD, BFPDA, and PFPA) were similar to the reconstruction of the total number of IBs (Appendices S2–S6).

### 3.6 Correlations of IBs and ecological factors

The states of reconstructed IBs for two categories (epineurals and epipleurals; panel B‐C) and three regions (AFPD, BFPDA, and PFPA; panel D‐F), as well as three ecological factors (diet, water depth, and water velocity; panel G‐I), were mapped onto the character state tree of IBs based on the phylogenetic relationship (Figure 6). Some carnivorous fishes seem to have a greater number of IBs. For instance, the carnivorous species in the Danioninae, such as *Opsariichthys bidens* and *Raiamas guttatus*, had more IBs than the noncarnivorous species in this subfamily. The subfamily Cultrinae, where most of the species are recognized as carnivorous (Chen, 1998), had comparatively more IBs than most other subfamilies of cyprinids. In the subclade that including Cultrinae (Clade III‐2), these typically carnivorous fishes that had relatively more IBs are as follows: *Chanodichthys dabryi*, *Culter alburnus*, *Ochetobius elongatus*, and *Paralaubuca barroni*. Some subfamilies were omnivorous, such as Gobioninae; however, their IBs varied greatly. For instance, *Pseudorasbora parva*, *Saurogobio dabryi*, and *Abbottina rivularis* were three typical omnivorous fishes in the Gobioninae, but they had average IBs of 89, 136, and 94, respectively. We did not observe any correlations between IBs and water depth or water velocity. Moreover, some fishes have close phylogenetic relationships and similar habits, but have significantly different IB counts, that is, *Barilius caudiocellatus* (108) and *R. guttatus* (133).

## 4 DISCUSSION

The presence of IBs in fish muscles is something of a mystery to ichthyologists, as well as being a troublesome issue in aquaculture—especially in countries like China where cultivation of cyprinids plays an important role in people's nutrition (Tang, Han, Mao, Zhang, & Shan, 2016). The unpredictable numbers and positions of IBs when eating a fish impact people's willingness to pay a good price for cyprinids in fish markets. An understanding of IBs has several potential practical applications. In this work, we systematically examined the issues involving IBs based on a large sample of cyprinids from a phylogenetic viewpoint.

A reliable and repeatable method of counting IBs is fundamental to their study. Up to now, three methods have been used: anatomy, skeletal staining, and X‐ray photography. Anatomy and skeletal staining are applicable to nearly every laboratory because no special equipment is needed; however, these methods involve damage to the samples. Additionally, anatomy, despite being straightforward, can prove difficult when dealing with small individuals, while skeletal staining requires a large amount of processing time. Compared to the above two methods, using X‐ray photograph to count IBs is a rapid and noninvasive technique, and has thus become a very convenient and popular approach recently (Li et al., 2013; Perazza et al., 2017; Vallod & Arthaud, 2009; Xu et al., 2015). In this study, we compared IB counts between anatomy and X‐ray photographs based on 20 medium‐sized individuals of *S. grahami*. We confirmed that the results of X‐ray photographs were not significantly different from those of anatomy (Figure 2). In consideration of the many advantages of X‐rays, such as ease of operation, time‐saving, no specimen damaging, and especially its applicability to living fishes, we propose that X‐ray photographs are superior to the other two methods. The only drawback is the availability of an X‐ray machine.

Three categories of IBs were recognized, namely epineural, epipleural, and epicentral (Owen, 1866). There has been a debate about whether cyprinids have all the three kinds of IBs or not. Dong et al. (2006) in a study of the IBs of *H. molitrix*, *H. nobilis*, *M. amblycephala*, and *Carassius gibelio* claimed that all the examined fishes had three categories of IBs. However, later studies showed the fishes mentioned in Dong et al. (2006) had only epineurals and epipleurals, but no epicentrals (Li et al., 2017; Lv et al., 2007; Wan et al., 2016). In our work, based on the large number of specimens and species that we examined, we confirm that epicentrals are not present in cyprinid fishes.

The IB counts in this study reveal that the total number of IBs in cyprinids ranged from 73 to 169 (Figure 3). This range is greater than that reported in a previous study by Lv et al. (2007), which we attribute to the more extensive material examined in our study. The IB counts of most species were similar to those reported by previous researches (Dong et al., 2006; Jiang, Yang, & Bao, 2008; Ke et al., 2009; Li et al., 2013; Lv et al., 2007), which suggests that the IBs are relatively stable at the species level. For instance, in this study, the sample size of each species was generally more than 3 and the proportion of intraspecific deviations of IBs were usually <8% (Appendix S1). However, the IBs of artificially bred species seem to be more complicated. The only species sampled from fish farm in this study, *S. grahami*, showed a wide range of intraspecific deviation (21%). This intraspecific instability was also found in the different strains of artificially bred common carp: There was a significant difference (*p* < 0.001) among three examined strains, and these differences were found mainly in the posterior regions of the fish (Cao et al., 2014). In dividing the fish body into three regions (Figure 1), we also found that the number of IBs in PFPA was generally greater than those in AFPD and BFPDA, and in some species, these differences could be as great as five times the normal number. Therefore, we suggest that the PFPA region might be the optimal target for selective breeding for fewer IBs in cyprinids.

The number of sarcomeres reflects, to some extent, the number of vertebrae in fishes (Chen et al., 2017). Intermuscular bones, which are usually located between two adjacent sarcomeres, supposedly correlate with the number of vertebrae. By counting the number of IBs and vertebrae from a large number of samples, we confirm that IBs do have a positive correlation with vertebrae in cyprinid fishes with a correlation coefficient of 0.802 (Figure 4). Therefore, it is possible to make a preliminary estimation of which of two fishes has the greater number of IBs by extrapolating from their number of vertebrae.

To the authors' knowledge, this paper is the first attempt to investigate the possible evolutionary patterns of IBs from a phylogenetic perspective. To achieve this goal, a strongly corroborated phylogenetic tree was indispensable. In this study, phylogenetic relationship reconstruction of the Cyprinidae was obtained based on three mitochondrial DNA fragments (CYT *b*, COI, and ND4). The tree topology (Figure 5) was largely consistent with most of the previous genetic studies derived from different DNA fragments (Wang et al., 2012; Yang, Sado, et al., 2015). The three major subclades (Clades I, II, and III) were also identical with those found by Jiang et al. (2018), who provided a well‐resolved inter‐subfamily relationship of cyprinids at genome level (although the monophyly of each subfamily was beyond their aims for that paper). In this study, discussion of the deep phylogenetic relationship of the Cyprinidae is not our main focus; however, the tree we present here indicates that most of the morphological‐based subfamilies proposed by Chen (1998) are not monophyletic groups (Figure 5), thus implying this popular 12‐subfamily classification (Chen, 1998) needs to be revised. Based on the phylogenetic tree in this study (Figure 5), the number of IBs seemed to have phylogenetic relevance. For instance, the species in Clade III‐2, including the nominal subfamilies Hypophthalmichthyinae, Xenocyprinae, and Cultrinae and some species of Leuciscinae and Gobiobotinae, turned out to have similar numbers of total IBs, epineurals, and epipleurals (Figure 6). *Tinca tinca,* a species previously placed in the Leuciscinae, has long been treated as *incertae sedis* in both morphological and molecular studies (see more discussion in Jiang et al., 2018). Here, this species had a similar IB composition to *Acheilognathus*, but distinct from most other Leuciscinae species (Figure 6). The close relationship of *Tinca tinca* and the Acheilognathinae revealed in this study (Figure 5) was also demonstrated by Yang, Sado, et al. (2015) based on both mitochondrial and nuclear DNA fragments. *Percocypris*, previously placed in the Barbinae (Chen, 1998), was revealed in our tree as the sister group of *Schizothorax*, which is consistent with a previous molecular study that suggested *Percocypris* originated from a common ancestor with *Schizothorax* (Wang, Yang, & Chen, 2013). No matter which categories of IBs were counted, those in *Percocypris* and *Schizothorax* were similar in having high numbers relative to other species in the Barbinae. In spite of the above correlation between phylogenetic relationships and IBs, the major lineages did not display distinguishable phylogenetic patterns, as some species with close relationships had divergent IB numbers. Thus, we speculate that although some of the evolution of IBs was under a phylogenetic framework, the number of IBs was varying frequently due to other internal or external (nonhistorical) factors.

Ecological factors, such as diet, water depth, and water velocity, could be reasons affecting the evolution of IBs in terms of speciation and adaptation. In this study, we mapped the three ecological factors onto the phylogeny to investigate whether there are any correlations between IBs and these factors. It would appear that some carnivorous fishes have more IBs, which was reflected from a comparison of some carnivorous and noncarnivorous species in the Danioninae, and in the whole subfamily of Cultrinae. The possible explanation is that carnivorous fishes usually need to accelerate very rapidly when starting to catch for prey after switching from ambush status. This would require more IBs in the posterior region of the body, if IBs do indeed transmit and enhance muscle strength as previous studies have suggested (Danos & Staab, 2010; Dong et al., 2006; Lv et al., 2007). However, when we looked at omnivorous fishes, the situation was more complicated. For example, the closely phylogenetically related species, *P. parva*, *S. dabryi*, and *A. rivularis*, displayed significantly divergent IB numbers. We did not observe any correlation between IBs and water depth or velocity. We infer that the evolution of IBs in the Cyprinidae would probably not be affected by single ecological factor, but rather be a product of multiple factors. For instance, we compared the number and shape of IBs in *Sinocyclocheilus*, a group that is characterized by containing many cave species. We found the typical cave‐dwelling species (known as troglobites) had fewer IBs than those atypical cave species (known as troglophiles, see also in Yang, Pan, et al., 2015). Caves are an extreme environment without light, which embody a series of different ecological factors relative to open water (e.g., scarce of food supply, low water fluctuation, and low predation pressure).

In summary, the variations of IBs in the modern Cyprinidae were full of diversity, which may reflect their phylogenetic history, but has then been shaped by multiple environment factors.

## CONFLICT OF INTEREST

The authors declare that they have no conflicts of interest to this work.

## AUTHOR CONTRIBUTIONS

KFY, WSJ, and JXY designed the work and scientific objectives. KFY, XAW, YWZ, and XFP collected and prepared the fish samples. KFY analyzed the data. KFY and WSJ prepared the manuscript. JXY revised the manuscript. All authors have read and approved the final manuscript.

## Data Availability

DNA sequences: All sequences used in this study were downloaded from NCBI (see accession numbers in Appendix S1). The following datasets have been deposited in Dryad http://doi.org/10.5061/dryad.3jt83k4. Dataset **S1** **.** DNA sequence alignment for the concatenated dataset with three genes in NEXUS format. Dataset **S2**. Input file for Bayesian inference (BI) analyses of the concatenated dataset in NEXUS format. Dataset **S3**. Input file for Mesquite analysis in NEXUS format.
