# Supplementary material for: Evolution of the intermuscular bones in the Cyprinidae (Pisces) from a phylogenetic perspective
Source: Ecol Evol. 2019 Jul 17;9(15):8555–66. doi: 10.1002/ece3.5374 (PMC6686301; doi:10.1002/ece3.5374)

| **Appendix S1. IBs counts and sequence information of species used for phylogenetic relationship reconstruction in this study.** | | | | | | | | |
| --- | --- | --- | --- | --- | --- | --- | --- | --- |
| Subfamily name | Species | IBs counts information | | |  | GenBank accession numbers | | |
|  |  | Sample size | TN of IBs | Proportion of D-value |  | CYT *b* | COI | ND4 |
| Danioninae | *Barilius caudiocellatus* | 9 | 104-110 | 5.45% |  | HM224319 | KM610430 | / |
|  | *Opsariichthys bidens* | 8 | 103-112 | 8.04% |  | AY646649 | HM224195 | DQ367044ª |
|  | *Raiamas guttatus* | 9 | 130-135 | 3.70% |  | HM224331 | HM224214 | NC_015547ª |
|  | *Yaoshanicus arcus* | 3 | 95-96 | 1.04% |  | AF309086 | NC_015540ª | NC_015540ª |
|  | *Zacco platypus* | 6 | 109-116 | 6.03% |  | AY245089 | EF452896 | EF452825 |
| Leuciscinae | *Ctenopharyngodon idella* | 5 | 114-119 | 4.20% |  | HM238043 | KR861836 | EU391390ª |
|  | *Ochetobius elongatus* | 1 | 141 | 0.00% |  | AF309506 | NC_025646ª | NC_025646ª |
|  | *Rhynchocypris lagowskii* | 2 | 101-110 | 8.18% |  | EF094550 | HQ536405 | KR091310ª |
|  | *Squaliobarbus curriculus* | 7 | 109-114 | 4.39% |  | HM224308 | HM224189 | KC351187ª |
|  | *Tinca tinca* | 2 | 100-103 | 2.91% |  | HM167957 | HQ961062 | NC_008648ª |
| Cultrinae | *Chanodichthys dabryi* | 4 | 129-133 | 3.01% |  | KC526217ª | KC526217ª | KC526217ª |
|  | *Culter alburnus* | 8 | 129-139 | 7.19% |  | KM044500ª | KR861849 | KM044500ª |
|  | *Hemiculter leucisculus* | 9 | 119-125 | 4.80% |  | AF494362 | KF492989 | NC_022929ª |
|  | *Hemiculterella macrolepis* | 3 | 121-124 | 2.42% |  | EF151094 | / | / |
|  | *Metzia lineata* | 6 | 91-95 | 4.21% |  | KJ585805 | HM224187 | AP011220ª |
|  | *Paralaubuca barroni* | 8 | 126-131 | 3.82% |  | KF029697 | KF029681 | / |
|  | *Pseudohemiculter dispar* | 7 | 128-137 | 6.57% |  | KF029700 | KF029684 | NC_020435ª |
|  | *Sinibrama macrops* | 7 | 120-124 | 3.23% |  | NC_020013ª | NC_020013 ª | NC_020013ª |
| Xenocyprinae | *Distoechodon tumirostris* | 8 | 127-134 | 5.22% |  | AF036175 | GU434343 | AF036179ª |
|  | *Xenocypris hupeinensis* | 3 | 112-117 | 4.27% |  | AF036165 | GU434330 | AF036177ª |
|  | *Xenocypris macrolepis* | 8 | 109-117 | 6.84% |  | HM224310 | GU434328 | AF036185ª |
| Hypophthalmichthyinae | *Hypophthalmichthys molitrix* | 4 | 116-118 | 1.69% |  | AF051866 | JX983319 | HQ235870 |
|  | *Hypophthalmichthys nobilis* | 4 | 109-116 | 6.03% |  | JQ346141 | JX260831 | HQ235871 |
| Gobioninae | *Abbottina rivularis* | 6 | 92-96 | 4.17% |  | AY953020 | KR861747 | KM081703ª |
|  | *Hemibarbus maculatus* | 6 | 114-124 | 8.06% |  | AY952990 | KR861931 | NC_018534ª |
|  | *Hemibarbus medius* | 5 | 116-121 | 4.13% |  | AY952989 | NC_024527ª | NC_024527ª |
|  | *Platysmacheilus exiguus* | 6 | 103-108 | 4.63% |  | AY953015 | NC_024163ª | NC_024163ª |
|  | *Pseudorasbora elongata* | 4 | 110-114 | 3.51% |  | AY952996 | KF245485ª | KF245485ª |
|  | *Pseudorasbora parva* | 1 | 89 | 0.00% |  | HM117901 | HQ961066 | JF802126ª |
|  | *Rhinogobio ventralis* | 5 | 120-131 | 8.40% |  | NC_022720ª | KR862162 | NC_022720ª |
|  | *Saurogobio dabryi* | 7 | 131-143 | 8.39% |  | AY245091 | JN003370 | KF612272ª |
|  | *Sarcocheilichthys nigripinnis* | 6 | 96-101 | 4.95% |  | KF013988 | JN003369 | NC_020608ª |
|  | *Sarcocheilichthys parvus* | 6 | 91-95 | 4.21% |  | EF193460 | NC_018786ª | NC_018786ª |
|  | *Sarcocheilichthys sinensis* | 5 | 94-100 | 6.00% |  | EF193417 | KC847084ª | KC847084ª |
| Gobiobotinae | *Gobiobotia abbreviata* | 3 | 103-108 | 4.63% |  | AF051861 | / | / |
|  | *Gobiobotia filifer* | 3 | 105-112 | 6.25% |  | AY953002 | NC_029187ª | NC_029187ª |
|  | *Gobiobotia kolleri* | 5 | 108-110 | 1.82% |  | NC_022931ª | NC_022931ª | NC_022931ª |
|  | *Gobiobotia meridionalis* | 9 | 105-113 | 7.08% |  | AF375867 | JN003344 | / |
| Acheilognathinae | *Acheilognathus chankaensis* | 3 | 87-92 | 5.43% |  | HQ113250 | NC_023101ª | NC_023101ª |
|  | *Acheilognathus macropterus* | 8 | 85-92 | 7.61% |  | EF571657 | KR861766 | KJ499466ª |
|  | *Acheilognathus tonkinensis* | 4 | 88-92 | 4.35% |  | EU636235 | / | / |
|  | *Rhodeus ocellatus* | 3 | 82-85 | 3.53% |  | AB769519 | DQ026430ª | DQ026430ª |
| Barbinae | *Acrossocheilus iridescens* | 5 | 99-105 | 5.71% |  | KP712196 | KP712096 | KP712317 |
|  | *Acrossocheilus kreyenbergii* | 5 | 90-98 | 8.16% |  | JX066772 | JX066754 | DQ244099ª |
|  | *Acrossocheilus stenotaeniatus* | 6 | 102-106 | 3.77% |  | NC_024934ª | NC_024934ª | NC_024934ª |
|  | *Acrossocheilus yunnanensis* | 5 | 115-119 | 3.36% |  | KC696535 | NC_028527ª | NC_028527ª |
|  | *Hypsibarbus vernayi* | 5 | 89-96 | 7.29% |  | HM536794 | HM536892 | HQ235785 |
|  | *Mystacoleucus marginatus* | 6 | 86-91 | 5.49% |  | HQ235762 | KU692644 | HQ235806 |
|  | *Neolissochilus benasi* | 4 | 112-118 | 5.08% |  | KC696527 | / | / |
|  | *Neolissochilus heterostomus* | 5 | 114-122 | 6.56% |  | AY463516 | / | / |
|  | *Onychostoma elongatum* | 4 | 117-121 | 3.31% |  | KP712152 | KP712049 | KP712268 |
|  | *Onychostoma gerlachi* | 4 | 117-121 | 3.31% |  | KC696548 | NC_026549ª | HQ235845 |
|  | *Onychostoma lepturum* | 5 | 116-119 | 2.52% |  | HM142578 | HM142528 | / |
|  | *Percocypris pingi* | 2 | 132-137 | 3.65% |  | JX042202 | JX042165 | JX316026ª |
|  | *Percocypris tchangi* | 4 | 123-134 | 8.21% |  | HQ235768 | HQ235973 | HQ235833 |
|  | *Poropuntius carinatus* | 2 | 109-119 | 8.40% |  | KC631278 | KC631186 | / |
|  | *Poropuntius huangchuchieni* | 5 | 110-112 | 1.79% |  | HQ235769 | / | HQ235807 |
|  | *Sikukia flavicaudata* | 5 | 95-100 | 5.00% |  | KC696538 | / | / |
|  | *Sinocyclocheilus angustiporus* | 3 | 111-117 | 5.13% |  | AY854702 | / | AY854759 |
|  | *Sinocyclocheilus anophthalmus* | 3 | 108-113 | 4.42% |  | AY854716 | NC_023472ª | AY854773 |
|  | *Sinocyclocheilus furcodorsalis* | 3 | 100-102 | 1.96% |  | HQ677332 | NC_019995ª | AY854766 |
|  | *Sinocyclocheilus grahami* | 50 | 90-114 | 21.05% |  | AY854696 | GQ148557ª | AY854753 |
|  | *Sinocyclocheilus guishanensis* | 2 | 114-120 | 5.00% |  | AY854722 | / | AY854779 |
|  | *Sinocyclocheilus hyalinus* | 1 | 92 | 0.00% |  | AY854721 | / | AY854778 |
|  | *Sinocyclocheilus jii* | 5 | 106-107 | 0.93% |  | AY854728 | / | AY854785 |
|  | *Sinocyclocheilus macrolepis* | 3 | 106-108 | 1.85% |  | AY854729 | / | AY854786 |
|  | *Sinocyclocheilus maitianheensis* | 3 | 102-110 | 7.27% |  | AY854710 | / | AY854767 |
|  | *Sinocyclocheilus malacopterus* | 3 | 105-118 | 11.02% |  | AY854700 | / | AY854756 |
|  | *Sinocyclocheilus multipunctatus* | 1 | 104 | 0.00% |  | AY854713 | / | AY854769 |
|  | *Sinocyclocheilus rhinocerous* | 3 | 92-100 | 8.00% |  | AY854720 | / | AY854777 |
|  | *Sinocyclocheilus tingi* | 8 | 104-112 | 7.14% |  | AY854701 | / | AY854758 |
|  | *Sinocyclocheilus yangzongensis* | 3 | 106-113 | 6.19% |  | AY854726 | / | AY854782 |
|  | *Spinibarbus denticulatus* | 4 | 102-113 | 9.73% |  | JX042205 | GU086582 | NC_021616ª |
|  | *Tor qiaojiensis* | 5 | 114-117 | 2.56% |  | GQ406316 | / | / |
|  | *Tor sinensis* | 5 | 109-114 | 4.39% |  | HM536802 | KT261297 | HQ235788 |
| Labeoninae | *Crossocheilus burmanicus* | 5 | 93-98 | 5.10% |  | JX074224 | GU086573 | JX074301 |
|  | *Discogobio brachyphysallidos* | 4 | 100-103 | 2.91% |  | GQ406319 | GU086585 | / |
|  | *Discogobio macrophysallidos* | 3 | 103-106 | 2.83% |  | GU086549 | GU086587 | / |
|  | *Discogobio tetrabarbatus* | 4 | 93-96 | 3.13% |  | KC696553 | GU086594 | JX074328 |
|  | *Discogobio yunnanensis* | 5 | 100-110 | 9.09% |  | GU086545 | GU086583 | NC_025319ª |
|  | *Garra orientalis* | 4 | 81-88 | 7.95% |  | JQ864585 | GU086602 | HM536702ª |
|  | *Garra qiaojiensis* | 3 | 89-92 | 3.26% |  | JQ864583 | JQ864604 | KF727438ª |
|  | *Pseudogyrinocheilus prochilus* | 5 | 116-122 | 4.92% |  | HQ235754 | GU086572 | HQ235800 |
|  | *Rectoris posehensis* | 4 | 113-115 | 1.74% |  | GQ406329 | GU086595 | HQ235796 |
| Schizothoracinae | *Gymnocypris potanini* | 6 | 120-136 | 11.76% |  | KC734002 | KU896808ª | KU896808ª |
|  | *Schizothorax dolichonema* | 2 | 126-127 | 0.79% |  | DQ126117 | HQ235952 | HQ235850 |
|  | *Schizothorax dulongensis* | 4 | 118-122 | 3.28% |  | AY954284 | HQ235968 | HQ235821 |
|  | *Schizothorax grahami* | 5 | 120-126 | 4.76% |  | HQ235774 | HQ235944 | HQ235843 |
|  | *Schizothorax kozlovi* | 3 | 130-134 | 2.99% |  | DQ126115 | HQ236011 | HQ235816 |
|  | *Schizothorax meridionalis* | 4 | 124-128 | 3.13% |  | AY954287 | KP712077 | KP712297 |
|  | *Schizothorax wangchiachii* | 6 | 128-133 | 3.76% |  | DQ126122 | HQ235943 | HQ235813 |
| Cyprininae | *Carassioides acuminatus* | 4 | 80-84 | 4.76% |  | LC097909 | LC097930 | AP011178ª |
|  | *Carassius auratus* | 2 | 78 | 0.00% |  | AB368694 | KJ874430ª | KJ874430ª |
|  | *Cyprinus carpio* | 4 | 91-95 | 4.21% |  | DQ868875 | KU050703ª | KU050703ª |
|  | *Cyprinus pellegrini* | 2 | 96-102 | 5.88% |  | JX042203 | JX042166 | / |
|  | *Cyprinus rubrofuscus* | 4 | 97-101 | 3.96% |  | KP712248 | KP712147 | KP712365 |
|  | *Procypris mera* | 2 | 105-107 | 1.87% |  | KC696555 | JX316027ª | JX316027ª |
| Nemacheilidae | *Homatula potanini* | 4 | 93-97 | 4.12% |  | JF340400 | KP749475ª | KP749475ª |
| Note: TN denotes the total number of IBs; D-value denotes the deviation value of IBs; slash (/) denotes missing data; superscript letter a (a) denotes that the gene sequence was extracted from the whole mitochondrial DNA sequence under that accession number. | | | | | | | | |

**Appendix S2. Ancestral reconstruction of the number of epineurals (EN).**

**
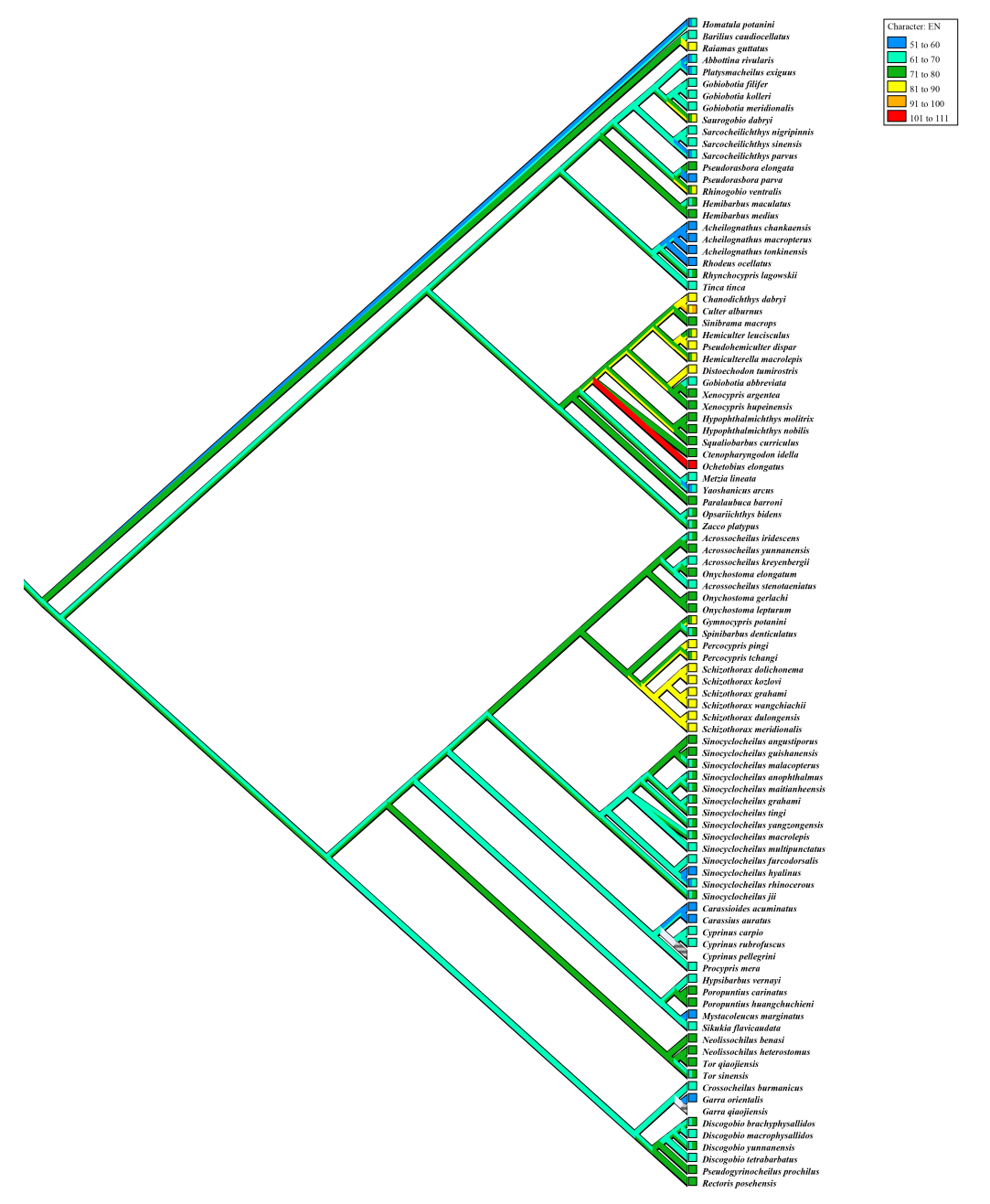
**

**Appendix S3. Ancestral reconstruction of the number of epipleurals (EP).**


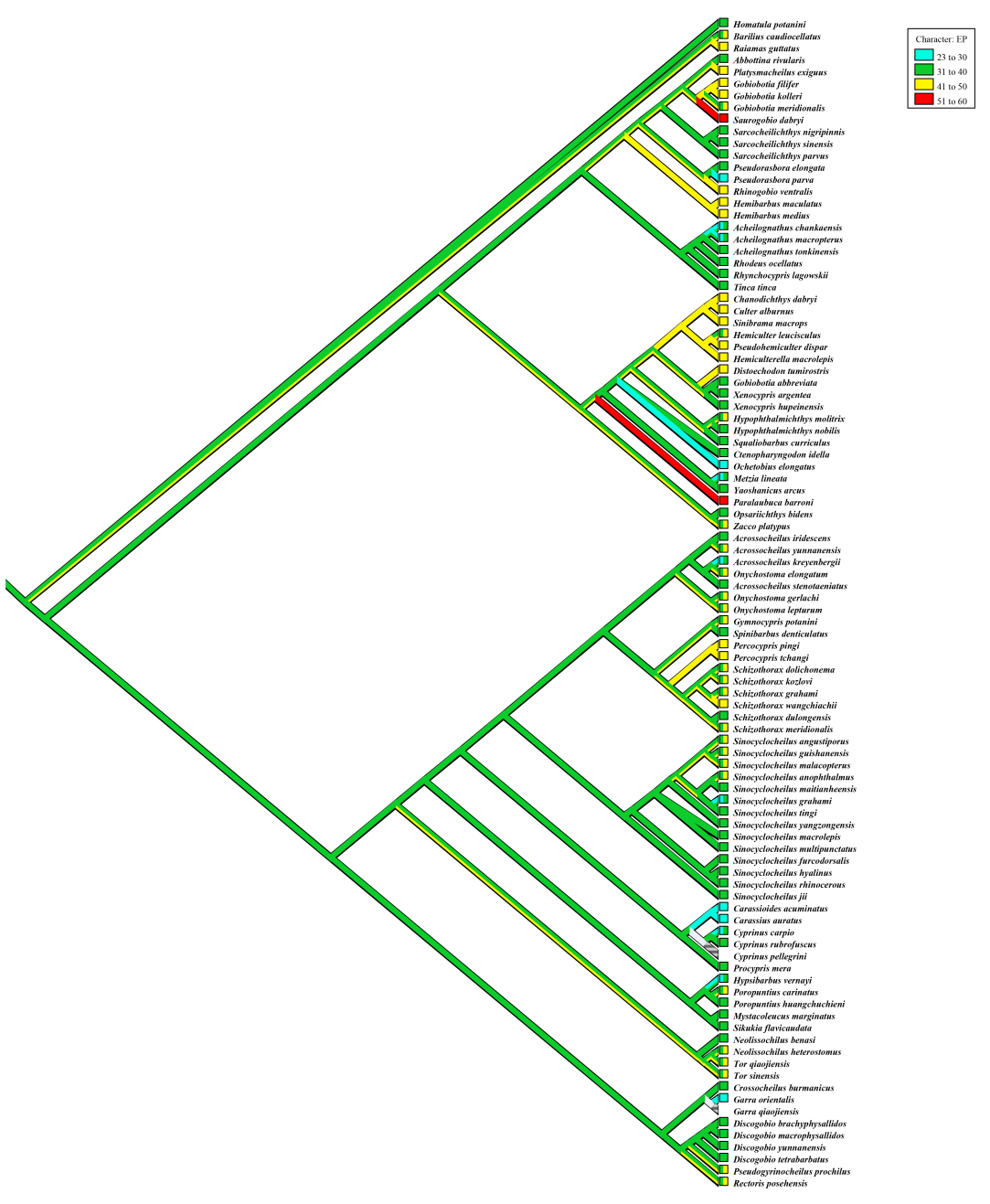


**Appendix S4. Ancestral reconstruction of the number from the region of anterior to the first ptereygiophore of the dorsal fin (AFPD).**


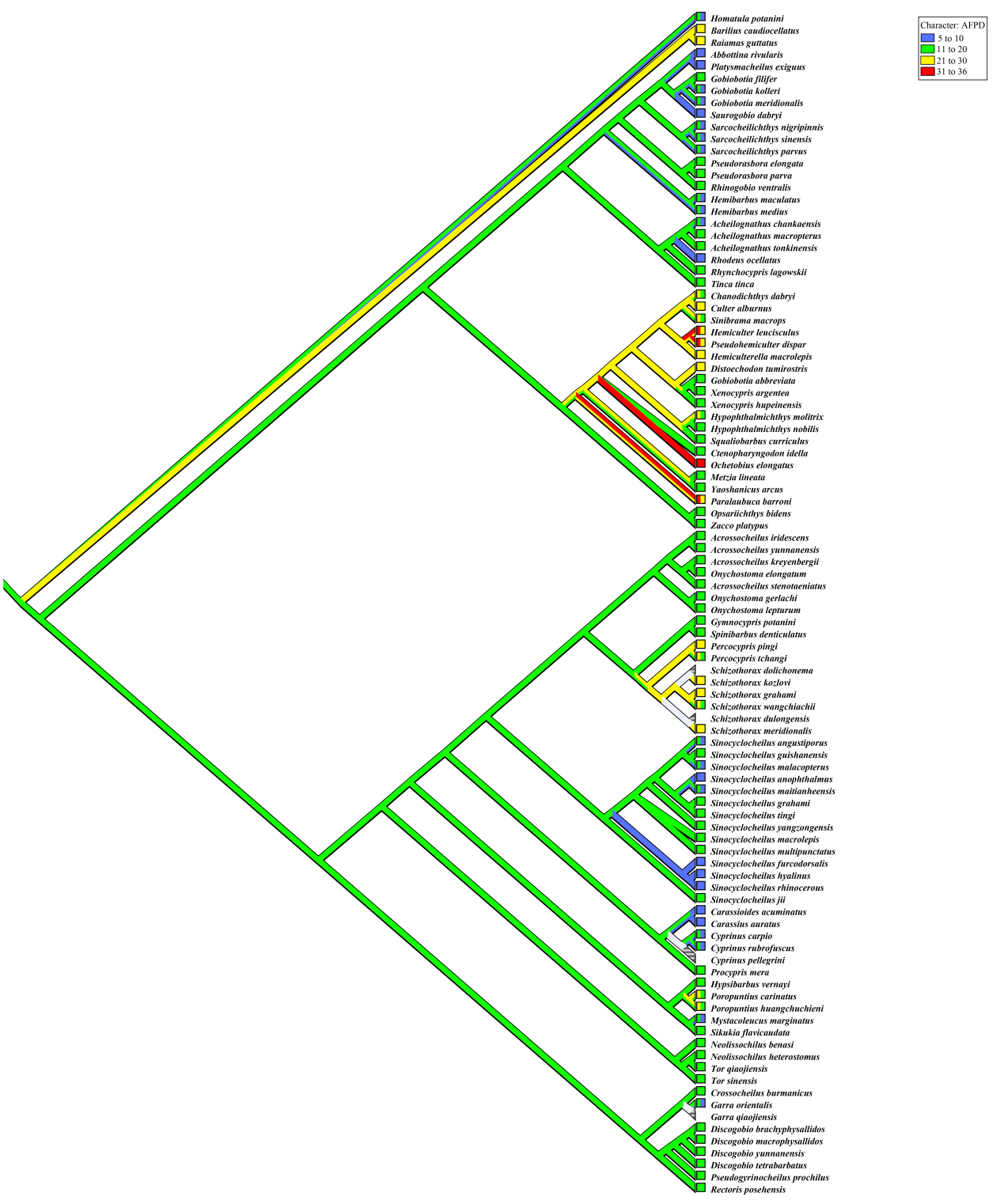


**Appendix S5. Ancestral reconstruction of the number from the region of between the first pterygiophore of the dorsal fin and the first pterygiophore of anal fin (BFPDA).**


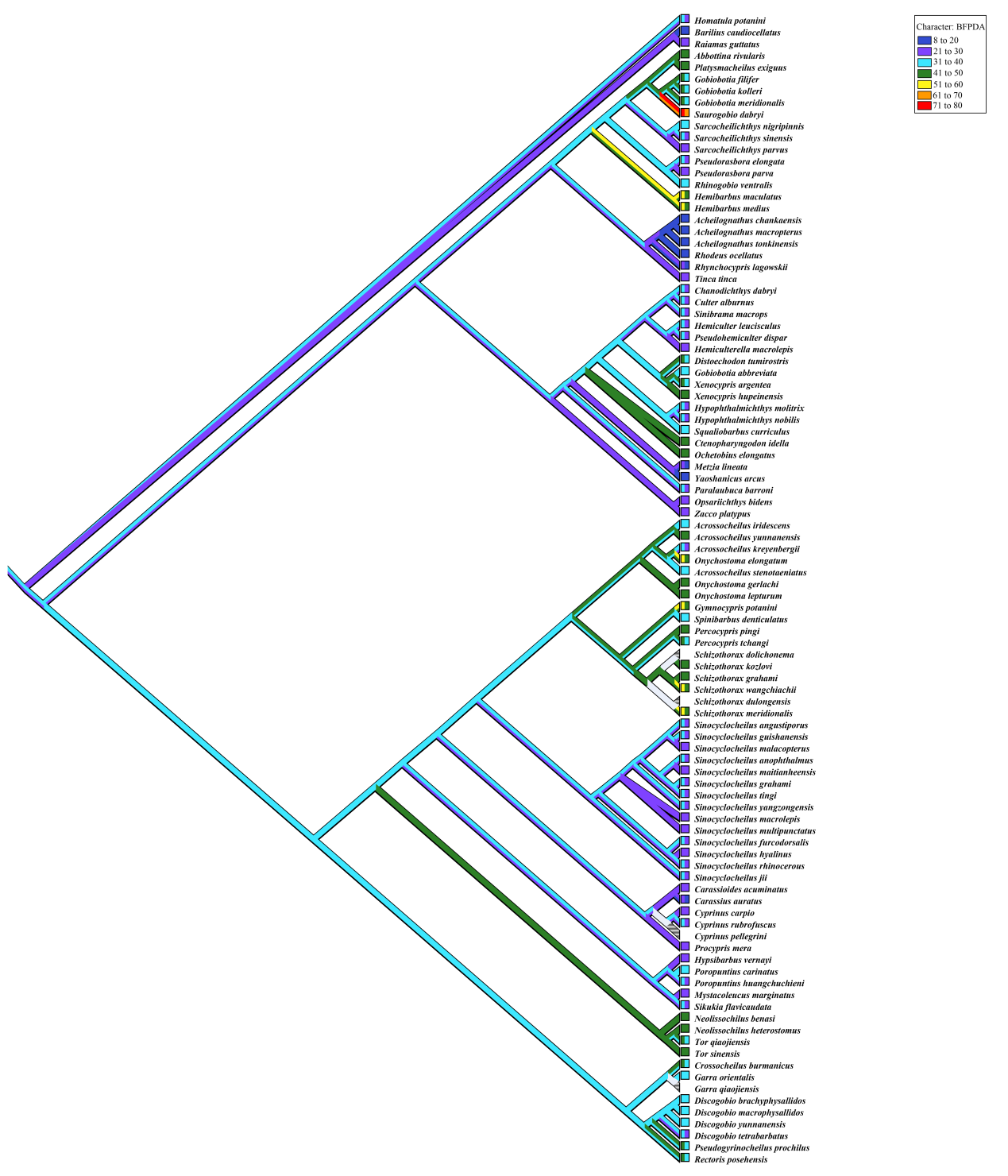


**Appendix S6. Ancestral reconstruction of the number from the region of posterior to the first pterygiophore of the anal fin (PFPA).**


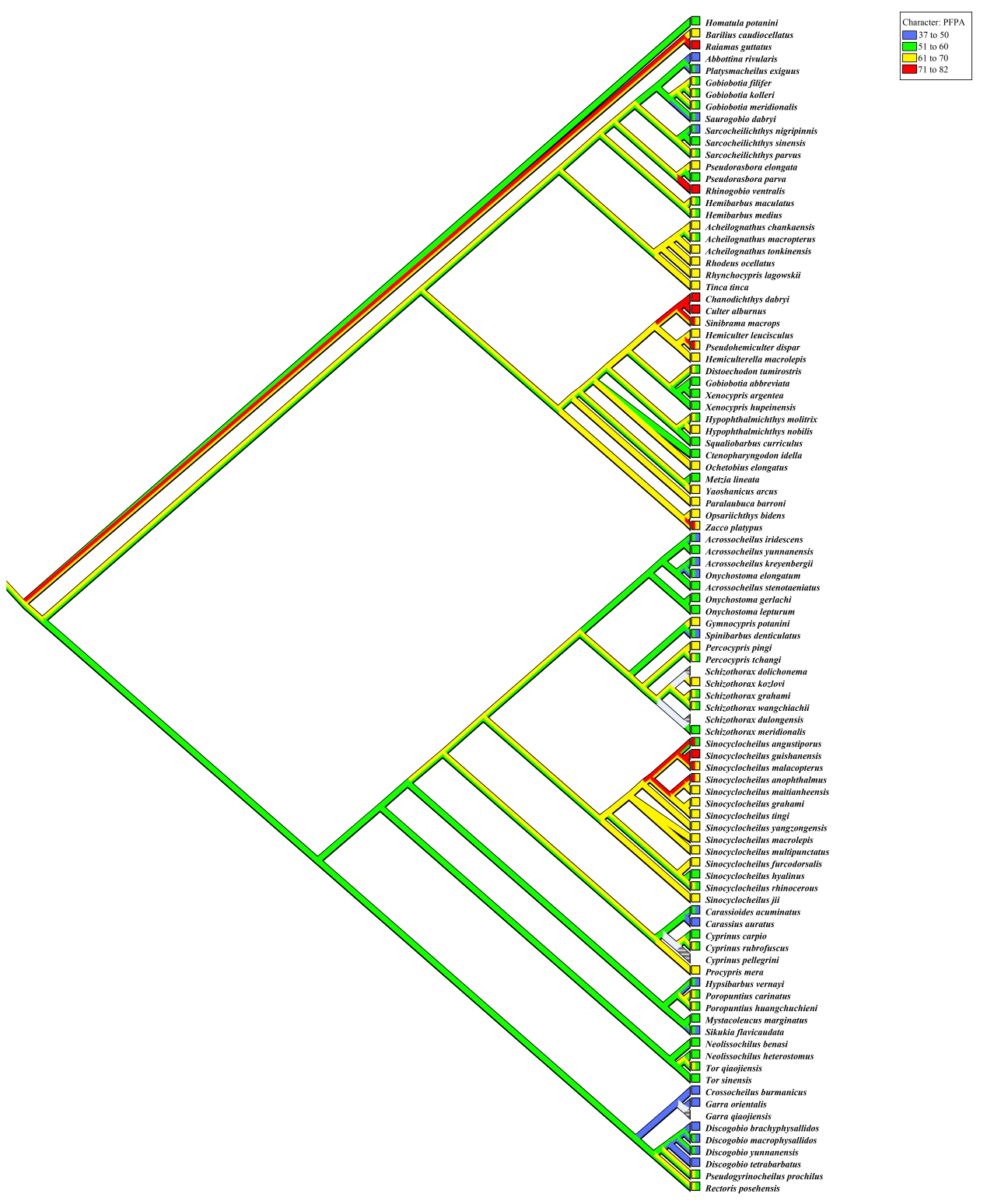

Supplement: Supplementary file 1 [file ECE3-9-8555-s001.docx]
